# British and Foreign Medical Review

**Published:** 1845-04

**Authors:** 


### BRITISH AND FOREIGN MEDICAL REVIEW.

One of the ablest periodicals in our language devoted to medicine is the British and Foreign Medical Review. No other review, we believe, is so often referred to by writers as authority. Its articles are uniformly written with care, and many of them display great ability. The character of the work is fully set forth in the advertisement on the cover of this Journal. We always read it with pleasure and instruction, and can recommend it to our professional brethren as a digest of current medical science and literature equal to any of the day. Few physicians have the ability to take all the excellent periodicals in medicine which it may be desirable to have an opportunity of reading, but we should suppose by a proper understanding among the members of the profession in any neighborhood, or by the formation of clubs, a number might be taken to which all might have access. Besides the numerous journals of our own country, the London Lancet, the Medico-Chirurgical Review, and the work which stands at the head of this paragraph, may all be procured with convenience, and would be read with great advantage by the practitioner. Y.

